# Reports on the Hospitals of the United Kingdom

**Published:** 1911-11-04

**Authors:** Henry Burdett


### REPORTS ON THE HOSPITALS OF THE UNITED KINGDOM.

By SIR HENRY BURDETT, K.C.B., K.C.V.O.

### LVI.—Boston Hospital, Lincolnshire.

It is thirty-two years since we first inspected the Cottage Hospital as it then was at Boston. Its situation, its plan and the park-like grounds in which it stood made it one of the most attractive of cottage hospitals. In the thirty-two years which have intervened the hospital has been extended on more than one occasion, but the additions have not always been made with wisdom, and the buildings as they stand to-day are inconvenient to administer, and reveal a disregard for the requirements of a nursing staff for the thirty beds which the hospital now contains that indicates a lack of apprehension on the part of someone in authority.

It is an interesting feature, as marking the stages of progression from a cottage to a larger hospital, that the strongest desire is expressed by the present executive staff to take out the dividing walls in the case of the four smaller wards, in order that the hospital may consist of large wards only for the reception of general patients. The private wards with two beds each for paying patients would be retained as at present under this scheme. But for these the wards would then be all large ones. The inconvenience of having the sanitary block—a recent addition and a good one structurally—placed at the extreme end of the building can be imagined. It must add greatly to the labour of nursing patients who are confined to bed, and be attended by manifest inconveniences which it is conceivable might outweigh the hygienic gain. In any case it is a striking proof of the disadvantages which arise from extensions made piecemeal, and not as part of a carefully-thought-out scheme from the first, for extending the hospital accommodation to meet the growing needs of an increasing population.

### Its Administrative Efficiency.

Few hospitals could display clearer evidence than the Boston Hospital affords of perfected administration due to the whole-hearted zeal and efficiency of the resident staff, and especially of the Matron and Sister-in-charge. Miss S. H. Stack and her assistant Miss A. M. Reindorp, both from University College Hospital, London, have shown since their appointment a few months ago what sterling hard work, backed by an up-to-date knowledge of what a hospital should be, can accomplish when pursued with zeal and devotion to duty. Miss Stack was away when we inspected this hospital on September 2, so that we had the advantage of putting her management to the strongest test, for during the holiday season slackness is apt to creep into the best hospitals unless the system is sound and the spirit of the chief administrator is strong enough to impress itself upon the whole staff, and especially upon the locum tenens in charge. Miss Reindorp answered satisfactorily every test, however severe, and having inspected the Lincoln County Hospital on the previous day the contrast between the actual condition administratively of the two Institutions was strikingly in favour of Boston Hospital. Excellent surgical work is done at Lincoln, which is blessed by at least one really eminent surgeon, but, however the medical staff of these two hospitals may compare, there is no doubt that administratively the Boston Hospital has been infinitely the more efficient of the two. Yet we regret to find that at Boston there seems to be an absence of apprehension of the efficiency of the present nursing staff, for no nurses' recreation room is provided, no beverages except water are allowed, not even milk, and the sleeping accommodation leaves much to desire, the bedsteads in more than one instance being too small and short to enable nurses of average height to sleep comfortably in them. Even the hygienic condition of some of these rooms was imperilled by the existence of a slop-sink in the wrong place which ought never to have been placed there. It should be removed without a moment's avoidable delay. Its removal should mean the provision of a hygienic an dproper slop-sink so as to avoid the labour and disagreeableness of having to carry slops, etc., down one or more flights of stairs. Another striking want of apprehension on the part of the Committee is the fact that, although the training of probationers is undertaken and the bulk of the nurses' work has to be done by probationers, not only is no salary paid to them in their first year, but the Committee insist upon each probationer paying the cost of their own washing, which is fixed at £2 12s. per annum each. No wonder great difficulty is experienced in obtaining suitable women to enter for training under such unusual conditions, having regard to the smallness of this hospital of thirty beds. The probationers receive £10 in their second year and £15 in their third year, *i.e.* about £7 10*s*. and £12 10*s*. net respectively. Truly a most inadequate remuneration in the circumstances. The discomforts of the nurses at the Boston Hospital will be manifest when we add that the only room in which they can have their food and spend their leisure time is the board room, and that the number of comfortable chairs for the nurses' use is less than half the number required; that on the days when the Committee meets nurses have nowhere to sit except their bedrooms. Yet, like all small hospitals, the efficiency of the administration depends mainly upon the efficiency and loyalty of the nursing staff, a fact which, in view of what we have already said, makes it imperative that the Committee should recognise this neglect of adequate provision for the nurses' comforts and accommodation, and resolve promptly to remedy the grievous and unnecessary drawbacks which at present attach to the life of a nurse in this hospital.

### The Condition of the Wards.

Several of the old wards have been converted into larger and better ones by the removal of party walls. These wards are bright, well lighted, airy and good. We consider that the party walls should be removed between the remaining small wards already referred to with as little delay as possible. The wards lack adequate equipment. They are covered with linoleum of various patterns not suitable for ward floors, which has become inefficient from wear and was in some wards full of holes. Either the whole of this linoleum should be removed and be replaced by suitable hospital linoleum of the best quality and strength, or, if the floors are good enough, they should by preference be polished. The patients' lockers are of an unhygienic and inferior type, and should be renewed by a better pattern without delay. The wards lack equipment in the sense that neither surgical trolleys nor adequate modern wagons with accommodation for the ward stores are anywhere provided. The bedding is defective from the fact that all beds have one and some more than one flock pillow or bolster. The reports issued during the last two years by the Local Government Board prove to demonstration upon incontrovertible evidence that no hospital, having due regard to hygienic considerations, should permit flock to be used for bedding in any form or shape. The basin in the corner of some of the wards for the use of the medical staff is so fitted as to render it imperative that the trap beneath each basin should be opened and cleansed once every week under a proper system of inspection. The cooking arrangements are of the simplest and most economical character, but produce results which from our inspection we conclude must give the maximum of satisfaction to the patients. One thing we noticed was that the refrigerators did not contain ice even in the hottest days of the present year, owing to the breakdown in the supply of this necessary commodity. We could elicit no adequate explanation of this breakdown, which would seem to arise from false economy somewhere, seeing that the Report expresses thanks to the Boston Deep Sea Fishing and Ice Company for fish and ice. Does this mean that the Committee allow a hospital so far as ice is concerned to be entirely dependent upon the free gifts of one company? If so, the sooner the Committee adopts a more businesslike system the better it will be for the efficiency of the hospital.

### Wanted—A Hospital Mortuary.

We inspected the so-called mortuary and found it to be little better than a shed, far too small for the requirements of thirty beds, where all post-mortems have to be performed under the worst possible conditions. This fact was clearly demonstrated by the presence of a body when we inspected this disgraceful accommodation. Disgraceful it certainly is in a Christian country, for it exhibits an absence of proper care and thought for the feelings of the patients' friends, a want of apprehension of the pathological requirements of a modern hospital, and a disregard for modern civilisation, which demands that the mortuary in these days shall be decently arranged with a separate post-mortem room, with a chapel or some reverently designed resting-place where the friends may see their dead in circumstances which display reverence and a tender regard for other people's feelings. In any case the Boston Hospital cannot permit the excellence of much which marks its administration to be longer detracted from by the absence of an adequate, up-to-date, suitable mortuary as an essential part of its equipment.

### General Conclusions.

This Hospital is on the whole beautifully kept, and the larger wards are most attractive. As a cottage hospital from the time it was built, thanks in no small degree to the zealous care and interest of the late Doctor Mercer Adam, it held a high place amongst Institutions of its type. Despite numerous additions, it deserves commendation for its efficiency in most respects, and is to-day in the hands of a resident staff who, when judged by their work, are most certainly proved efficient. It seems at the present time to lack the cordial continuous active support of the inhabitants of Boston, on whom the responsibility for a measure of personal service undoubtedly rests. The criticisms we have ventured to make point to a failure on the part of the Committee to apprehend the requirements of a modern up-to-date hospital which is blessed with an efficient staff. Everything which we have suggested is urgently required, and can be readily supplied at a relatively small cost. We rely upon the Committee to put these matters in hand without delay, and so to do their part to secure that the Boston Hospital shall be made a model of what a small voluntary hospital can and ought to be in the present day.

